# *Escherichia coli* Nissle 1917 Protects against Sepsis-Induced Intestinal Damage by Regulating the SCFA/GPRs Signaling Pathway

**DOI:** 10.3390/microorganisms12081622

**Published:** 2024-08-08

**Authors:** Yajie Wang, Huan Deng, Lin Xiao, Yisheng Pan

**Affiliations:** Department of Gastrointestinal Surgery, Peking University First Hospital, Beijing 100034, China; pkudocyoung@outlook.com (Y.W.); dh1366053033@163.com (H.D.); 15376732201@163.com (L.X.)

**Keywords:** *Escherichia coli* Nissle 1917, short-chain fatty acids, G protein-coupled receptors (GPRs), intestinal barrier

## Abstract

This study explores whether *Escherichia coli* Nissle 1917 (EcN) can preserve the integrity of the intestinal barrier by modulating the metabolism pathway of short-chain fatty acids (SCFAs) in a C57BL/6J mouse model of lipopolysaccharide (LPS)-induced acute enteritis and a model of a Caco-2 monolayer. The study involved establishing a septic shock model in mice through lipopolysaccharide (LPS) injection. Clinical scores and intestinal permeability were meticulously documented. Immunofluorescence was utilized to localize the tight junction proteins. A quantitative real-time polymerase chain reaction (qRT-PCR) was employed to assess the expression of G protein-coupled receptors (GPRs) signaling. Additionally, the supplement of acetate and butyrate with Caco-2 monolayers to elucidate the potential of EcN in augmenting the intestinal barrier primarily via the modulation of SCFAs and qRT-PCR was performed to detect the expression of tight junction proteins and the activation of the GPRs protein signaling pathway. EcN mitigated the clinical symptoms and reduced intestinal permeability in the colon of LPS-induced mice. It also enhanced the production of SCFAs in the gut and upregulated the expression of SCFA receptor proteins GPR41 and GPR43 in the colon tissue. Our findings reveal that EcN activates the SCFA/GPRs pathway, thereby preserving intestinal barrier function and alleviating inflammation in a mouse sepsis model.

## 1. Introduction

Sepsis after surgery represents a complex clinical condition stemming from a dysregulated host response to infection, leading to significant morbidity and mortality [1]. The intestinal barrier is severely compromised by septic shock, resulting in the release of a plethora of inflammatory mediators into the bloodstream. This can facilitate the translocation of bacteria and endotoxins from the gut into the circulation, exacerbating inflammatory reactions and further damaging the intestinal barrier. This vicious cycle accelerates disease progression. Consequently, preventing intestinal barrier breakdown is one of the foremost challenges following severe acute septic shock [2]. The pathological manifestations of acute septic shock include the disruption of tight junctions in the gut epithelium and dysbiosis of the intestinal flora [3].

A nonpathogenic Gram-negative strain *Escherichia coli* Nissle 1917 (EcN) has been found to be used to treat patients with digestive disorders [4,5]. The mechanism underlying EcN’s beneficial properties involves the secretion of proteins that alter the expression or distribution of tight junction (TJ) proteins [6] and exert a regulatory influence on gut microbiota [7,8], thereby preserving intestinal barrier integrity. Our previous studies have confirmed EcN’s protective effects against intestinal barrier injury induced by the incurrence of sepsis [9,10,11].

Short-chain fatty acids (SCFAs), comprising acetic, propionic, and butyric acids, among others, are common metabolites of the gut microbiota. SCFAs are primarily produced in the colon from undigested carbohydrates and can also arise from protein fermentation [12]. SCFAs serve as the primary energy source for intestinal epithelial cells and are crucial for maintaining the intestinal mucosal barrier. They also stimulate the production of antimicrobial peptides, such as lysozyme, defensins, and mucins; enhance the secretion of these peptides; and bolster intestinal immune function [13]. As ligands for G protein-coupled receptors (GPRs), SCFAs can activate anti-inflammatory signaling cascades and maintain intestinal homeostasis. GPR41 and GPR43 are specific receptors for SCFAs and are pivotal in regulating metabolic disorders and immunity [14,15,16].

However, it remains unclear whether EcN can be utilized to treat sepsis by modulating intestinal microbiota levels through the SCFA/GPRs pathway. Consequently, we investigated whether EcN could preserve intestinal barrier integrity by regulating SCFA metabolism in a mouse model of LPS-induced acute enteritis and a Caco-2 monolayer epithelium model.

## 2. Method

### 2.1. Animals and Experimental Design

Thirty-six healthy 8-week-old male C57BL/6J mice, weighing between 25 and 30 g, were sourced from the Laboratory Animal Center of Peking University First Hospital (Beijing, China) and maintained under specific pathogen-free conditions. Following a 7-day acclimatization period, the mice were randomly allocated into four groups: control, LPS, EcN, and EcN + LPS, with each group consisting of nine mice. If the mice had an adverse event (death or excessive stress) during modeling, they were excluded from the study. The mice were modeled in such a way that all conditions were as similar as possible to avoid confounding factors.

**Figure 1:**
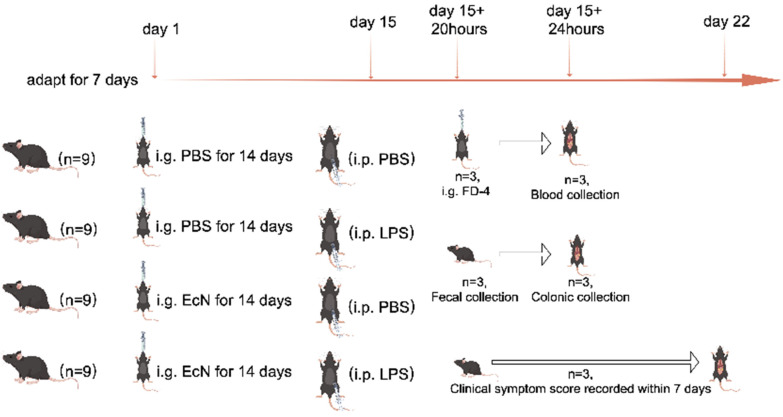
The detailed animal testing process. i.g., intragastric administration; i.p., intraperitoneal injection.

Our study markers were recorded from the first day of intragastric administration of PBS or EcN bacterial solution in mice. For the control and LPS groups, the mice were administered PBS via intragastric gavage, while the control + EcN and septic shock + EcN groups received EcN (10^9^ CFU/mL) daily for 14 days. On the 15th day, mice in the LPS and LPS + EcN groups were injected intraperitoneally with LPS at 5 mg/kg body weight, while the control and EcN groups received PBS with equivalent volume intraperitoneally. Twenty hours after intraperitoneal injection of PBS or LPS, three random mice in each group were given FD-4 gavage and labeled. Four hours later, these three mice were euthanized and used for obtaining eye blood to calculate FD-4 flux sampling; another three mice in each group were euthanized to obtain colon and intestinal feces. The remaining three mice in each group were retained to record changes in clinical status scores over a seven-day period. Finally, all mice were then euthanized with 3% pentobarbital sodium after the whole experiment. The detailed animal testing process can be seen in Figure 1.

Animal studies must be reported by the ARRIVE guidelines [17]. In different stages of the experiment, the blind grouping and implementation were carried out by many people. In the process of modeling the experimental mice, attention was paid to reducing the pain of the mice. The study protocol was reviewed and approved by the Ethics Committee for Animal Experiments (No. 2022029).

### 2.2. Clinical Status and FD-4 Flux Measurement of the Intestinal Barrier of Mice

On the 16th day, blood serum was collected for intestinal permeability testing. The intestinal permeability was measured as described previously [18]. Briefly, after 4 h of starvation, mice received gavage with FITC-dextran 4000 Da (FD-4) (Sigma-Aldrich, St. Louis, MO, USA) solution (600 mg/kg). Then, mice were sacrificed 4 h later, and blood was collected by cardiac puncture and then separated by centrifugation. Plasm levels of fluorescence intensity were measured using a Synergy H2 microplate reader (Biotek Instruments, Winooski, VT, USA) (excitation, 490 nm; emission, 520 nm).

The clinical status of the mice was monitored for 7 days post-LPS administration, with the following parameters recorded: conjunctivitis (0–2), stool consistency (0–2), hair coat (0–2), and moderate stimulation (0–2).

### 2.3. Morphological Examination and Histopathological Analysis

The distal colons were fixed in 4% formalin for over 48 h. Subsequently, samples were prepared by rinsing under running water, dehydrating with gradient alcohol, and embedding with paraffin to generate sections that were 4 mm thick, which were stained with hematoxylin and eosin (H&E). The morphological alterations in the intestinal mucosa were scanned using a microscope. The inflammation intensity, inflammation degree, and crypt damage were used to rank colon histological damage, as previously described [19].

### 2.4. Immunofluorescence of Tight Junction Proteins in Animals

The proximal colons were embedded in an optimal cutting temperature (OCT) compound and sectioned into 5 μm slices, then deparaffinized and rehydrated. Antigen retrieval was performed using heat-mediated treatment with 0.01 mol/L sodium citrate buffer (pH 6.0). The slides were blocked with 0.1 mol/L PBS containing 10% normal goat serum. Primary antibodies against ZO-1 (1:100, Invitrogen, Waltham, MA, USA) and Occludin (1:100, Invitrogen, USA) were incubated at 4 °C overnight, followed by incubation with Alexa 488-coupled goat anti-rabbit (Invitrogen, USA) and Alexa 555-coupled goat anti-mouse (Invitrogen, USA) secondary antibodies for 1 h. Slides were stained with DAPI (4′,6-diamino-2-phenylindoles), and immunofluorescence images were captured using a Fluoview 1000 confocal microscope (Olympus, Tokyo, Japan) at 400× magnification [20].

### 2.5. 16S rRNA Sequence Analysis of Mouse Feces

In a barrier environment, the mice were placed in sterile cages and allowed to defecate naturally. The excreted feces were collected using sterile forceps, and then the samples were placed into 1.5 mL sterile EP tubes, ensuring proper labeling. Following collection, the samples were promptly stored at −80 °C for preservation. A minimum of six fecal pellets per mouse is required to ensure an adequate sample volume for analysis. 16S rRNA sequencing was conducted using the method below. In brief, DNA from the cecal contents of mice was extracted using a commercial DNA Stool Mini extraction kit (MagPure Universal DNA KF Kit, Berlin, Germany), adhering to the manufacturer’s guidelines. The V4 region of the 16S rRNA gene was amplified using the forward primer 515 F (5′-GTGCCAGCMGCCGCGGTAA-3′) and reverse primer 806 R (5′-GGACTACHVGGGTWTCTAAT-3′). Samples were barcoded and pooled to create the sequencing library. Paired-end 2 × 250 bp reads were generated on the Illumina MiSeq platform (Illumina, San Diego, CA, USA) and merged using FLASH (V1.1.11). Samples were assigned based on their unique barcodes. High-quality clean tags were obtained under specific filtering conditions and clustered into operational taxonomic units (OTUs) using USEARCH with a 97% pairwise identity threshold. OTU representative sequences were taxonomically classified using the Greengenes database by the Ribosomal Database Project (RDP) Classifier (V2.2). Alpha diversity was analyzed using Mothur (V1.41.0), and beta diversity was assessed using QIIME (V1.9.0).

### 2.6. Determination of SCFAs Content

The stool of mice was determined and analyzed with liquid chromatography to determine the relative concentrations of SCFAs, including acetic acid, propionic acid, isobutyric acid, butyric acid, valerate acid, and isovalerate acid, following EcN administration. In brief, approximately 50 milligrams of freshly collected feces were taken and mixed with 0.5 milliliters of water, followed by ultrasonic agitation for 10 min. Subsequently, 0.5 milliliters of internal standard solution was added, and the mixture was vortexed for 30 min to facilitate extraction. The sample was then centrifuged at 4 °C and 12,000 revolutions per minute (rpm) for 5 min. Then, 50 microliters of the supernatant were collected and combined with 50 microliters of isotopically labeled internal standard (5 micrograms per milliliter). Additionally, 50 microliters of 3-nitrophenylhydrazine (3-NPH) solution (250 millimolar, prepared in a 50% methanol/water solution) and 50 microliters of 1-ethyl-3-(3-dimethylaminopropyl)carbodiimide (EDC) solution (150 millimolar, prepared in a 75% methanol/water solution containing 7.5% pyridine, resulting in a ratio of methanol:water:pyridine = 69.375:23.125:7.5) were added. The mixture was then placed on a shaker and allowed to react at 30 °C for 30 min. Afterward, 50 microliters of butylated hydroxytoluene (BHT) methanol solution (2 milligrams per milliliter) and 250 microliters of 75% methanol/water solution were introduced. The centrifuge tube was then placed in a low-temperature centrifuge and spun at 4 °C and 12,000 rpm for 5 min. Then, 100 microliters of the supernatant were taken and placed in an injection vial for mass spectrometry detection. The chromatographic column used was a Waters UPLC BEH C8 column (100 mm × 2.5 mm × 1.7 μm).

### 2.7. Cell Culture

The human intestinal epithelial cell line Caco-2, sourced from the American Type Culture Collection (ATCC), was cultured in Dulbecco’s Modified Eagle Medium (DMEM), supplemented with 10% fetal bovine serum (FBS) and 1% penicillin-streptomycin (P/S). Cultivation occurred within a CO_2_ incubator set at 37 °C and 5% CO_2_. Caco-2 monolayers are representative of colon cells. To obtain an intestinal barrier model, we had to polarize the cells on filters for 2–3 weeks. After the successful construction of the colonic epithelial model, the cells were divided into four groups: the control group, LPS group, Acetate + LPS group, and Butyrate + LPS group. The latter three groups were transferred to a medium containing 10 ng/mL LPS. Then, 24 h later, acetate and butyrate with concentrations of 5 mM were added to the Acetate + LPS group and Butyrate + LPS group, respectively.

### 2.8. Quantitative Reverse Transcription Polymerase Chain Reaction (qRT-PCR)

According to the instructions, RNA was extracted using TRIzol (Invitrogen) RNA extraction protocol, and subsequently, a high-capacity cDNA reverse transcription kit (TIANGEN, #KR116) was used for cDNA synthesis. Real-time quantitative PCR was performed using SYBR Green PCR reagent (TIANGEN, #FP205) using the following PCR cycle parameters: 95 °C for 15 min followed by 40 cycles of 95 °C for 10 s, 55 °C for 30 s, and 72 °C for 30 s with final extension at 72 °C for 10 min. GAPDH was used as a control for comparison. The primer sequences for the target genes were as follows: ZO-1 (forward, 5′-ACCACCAACCCGAGAAGAC-3′; reverse, 5′-CAGGAGTCATGGACGCACA-3′), Occludin (forward, 5′-TTGAAAGTCCACCTCCTTACAGA-3′; reverse, 5′-CCGGATAAAAAGAGTACGCTGG-3′), Claudin-1 (forward, 5′-GGGGACAACATCGTGACCG-3′; reverse, 5′-AGGAGTCGAAGACTTTGCACT-3′), GPR43 (forward, 5′-TGCTACGAGAACTTCACCGAT; reverse, 5′-GGAGAGCATGATCCACACAAAAC), GPR41 (forward, 5′-TTCACCA CCATCTATCTCACCG; reverse, 5′-GGAACTCCAGGTAGCAGGTC). GAPDH (forward, 5′-CAAGTTCAACGGCACAG-3′; reverse, 5′-CCAGTAGACTCCACGACAT-3′) serves as an internal control.

### 2.9. Statistical Analysis

The exact value of n in each experimental group is reported for each analysis. A two-tailed Student’s *t*-test (unpaired) was used to analyze differences in mean values between groups (GraphPad Prism version 10.0 for Macbook, GraphPad Software, Boston, MA, USA). A *p*-value < 0.05 was used to indicate the statistical significance. All results are expressed as the means ± standard error of the mean (SEM).

## 3. Results

### 3.1. LPS-Induced Acute Septic Shock in Mice

**Figure 2:**
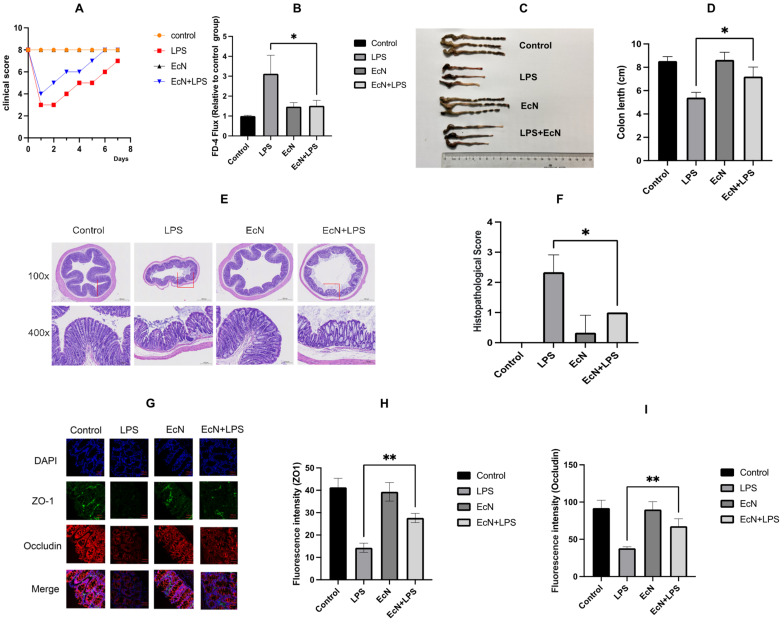
(**A**) The clinical status score after modeling. (**B**) Plasma FITC-dextran 4000 Da (FD-4) concentration. (**C**,**D**) Macroscopic pictures of the colon. (**E**,**F**) H&E staining of the colon. (**G**–**I**) Immunofluorescence of tight junction proteins. DAPI (blue), ZO-1 (green), Occludin (red). Control, control group; LPS, LPS group; EcN, EcN group; EcN + LPS, EcN + LPS group. All data are shown as mean ± SEM (*n* = 3 for each group). * *p* < 0.05, ** *p* < 0.01, LPS versus EcN + LPS.

Following LPS administration for 24 h, the experimental mice exhibited characteristic signs of septic shock, such as progressive weight reduction, diarrhea, fecal occult blood, and a decline in clinical condition scores (Figure 2A). This observation confirms the successful establishment of a murine model of acute septic shock induced by LPS [21]. FD-4 fluorescence labeling experiments indicated that EcN significantly slowed the increase in intestinal permeability induced by LPS in mice (Figure 2B). Treatment with EcN was observed to mitigate the LPS-induced reduction in colon length and enhance the clinical condition scores. In addition, the colon length in the LPS-injected mice was markedly shorter than that of their counterparts in the control cohort (Figure 2C,D).

Histopathological results (Figure 2E,F) showed LPS-induced pathological changes in colon tissue. In the control group, the colonic tissue was in good condition, the structure of the submucosal plasma membrane and muscle layer was intact, the epithelial cells of the colonic mucosa were arranged in order, and there was no edema and inflammatory cell infiltration in the submucosa. Compared with the control group, the integrity of colonic tissue from the LPS group was destroyed, the epithelial tissue of the colonic mucosa was disorganized, the cells of the mucosal layer were necrotic and detached, the submucosal inflammatory cells were infiltrated, and the glandular structure was lost. EcN treatment significantly reduced colon damage in the EcN + LPS group.

Immunofluorescence assays revealed that the LPS group exhibited significantly diminished positive staining for tight junction proteins ZO-1 and Occludin when juxtaposed with the control group. In contrast, the EcN + LPS group demonstrated a pronounced increase in the expression of these proteins relative to the LPS group (Figure 2G–I). These results collectively suggest that EcN promotes the upregulation of tight junction proteins, thereby maintaining the integrity of the intestinal barrier.

### 3.2. EcN Increased the Number of Bacteria in the Gut That Produce SCFAs in Mice

**Figure 3:**
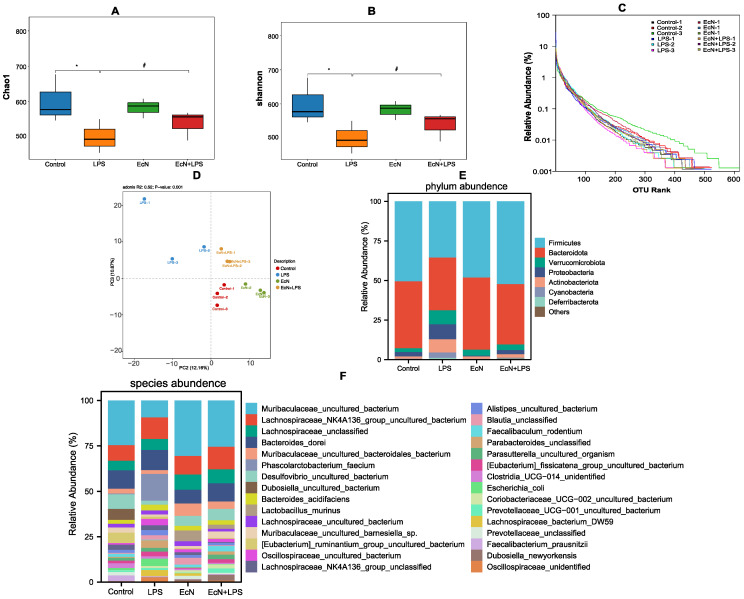
Effect of EcN on the gut microbiota of mice. Indicators of community diversity Chao1 (**A**) and Shannon (**B**). (**C**) Rank abundance curve in OTU level. (**D**) PLS-DA analysis in genus level. (**E**) Phylum abundance and (**F**) specifies abundance diversity between different groups. Control, control group; LPS, LPS group; EcN, EcN group; EcN + LPS, EcN + LPS group. Data are expressed as the means ± SEM (*n* = 3 for each group). * *p* < 0.05, LPS versus Control; # *p* < 0.05, LPS versus EcN + LPS.

16S rRNA sequencing was used to assess changes in the gut microbiota composition in different treatment groups. Our findings demonstrate that EcN modulates intestinal bacteria in LPS mice. The Chao1 and Shannon reflect the species richness of the community (Figure 3A,B). Compared with the control group, LPS significantly reduced the richness of microbiota in cecal contents, whereas the two indices were remarkably recovered when mice were pretreated with EcN. The rank abundance curve is often used to reflect species richness and community evenness, and the curve gradually tended to flatten, showing that the data of sample sequencing were logical and captured most of the flora diversity in all samples (Figure 3C). The PLS-DA results showed that EcN treatment shifted the intestinal flora toward the control group, while LPS treatment significantly altered the overall structure of the intestinal flora (Figure 3D).

We analyzed the differences in the relative abundance of the intestinal flora between different groups: Firmicutes and Bacteroidetes were the dominant phyla for all groups of intestinal microbiotas (Figure 3E). The relative abundance of *Firmicutes* and *Bacteroidetes* in LPS-treated mice was significantly reduced compared to the control group, while EcN was able to ameliorate the increased level of these two types of phylum abundance. The relative abundance of *Verrucomicrobiota*, *Proteobacteria*, and *Actinobacteroia* was increased in the LPS group, and pre-treatment of EcN could decrease them.

The results of the species-level analysis of the species composition map further revealed that (Figure 3F) while the proportions of Muribaculaceae, Lachnospiraceae_NK4A136_group, Lactobacillus in the LPS group were considerably decreased, their abundance increased following EcN pre-treatment.

**Figure 4:**
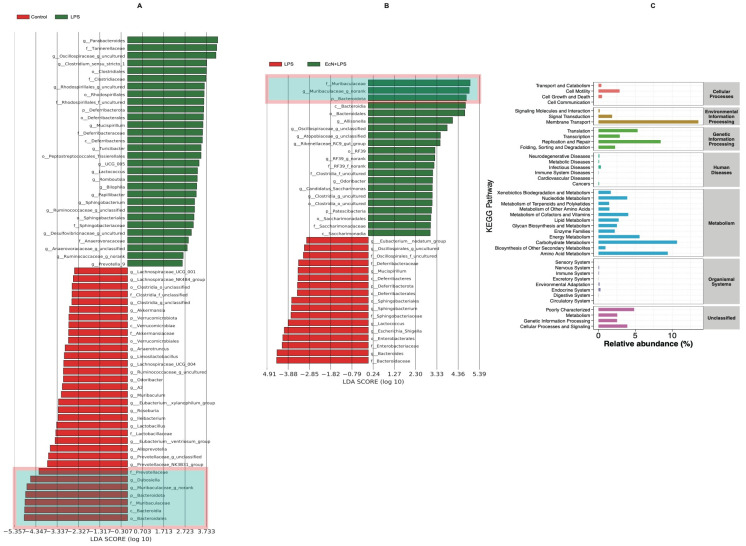
Effect of EcN on the gut microbiota of mice. Differentially abundant microbial taxa identified by LEfSe (**A**,**B**) with an LDA score. (**C**) KEGG pathway analysis based on different abundant microbiota. Control, control group; LPS, LPS group; EcN, EcN group; EcN + LPS, EcN + LPS group.

To further identify the crucial types of intestinal microbiomes among the different groups, LEfSe analysis was performed. As shown, *o_Bacteroidales*, *c_Bacteroidia*, *f_ f_Marinifilaceae*, *p_Bacteroidota*, *g_Muribaculaceae_g_norank*, *g_Dubosiella*, and *f_Prevotellaceae* were less abundant in LPS mice than in the control group. However, EcN pre-treatment promoted the accumulation of these six microbes and promoted the abundance of *f_Marinifilaceae*, *g_Muribaculaceae_g_norank*, *p_Bacteroidota*, and *o_Bacteroidales* (Figure 4A,B).

Figure 4C shows the KEGG signaling pathway enriched by intestinal differential bacteria in the LPS group and EcN + LPS group. The results show that EcN has obvious changes in cell motility, membrane transport, replication and repair, infectious diseases, and carbohydrate metabolism.

### 3.3. EcN Increased the Content of Short-Chain Fatty Acids Produced in the Gut of Mice and Increased the Expression of Short-Chain Fatty Acid Receptor Proteins GPR41 and GPR43 in Colon Tissue

**Figure 5:**
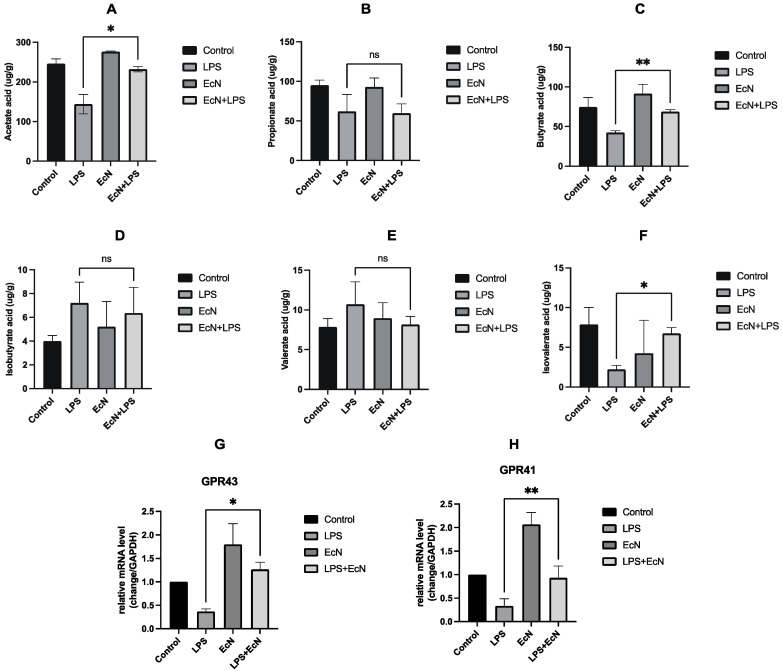
Effect of EcN on the content of SCFAs and GPRs in mouse feces. (**A**) acetic acid, (**B**) propionate acid, (**C**) isobutyric acid, (**D**) butyric acid, (**E**) valeric acid, and (**F**) isovalent acid. Effect of EcN on the expression of short-chain fatty acid receptor proteins, GPR43 (**G**) GPR41 (**H**) in mice colon. EcN, EcN group; EcN + LPS, EcN + LPS group. Data are expressed as the means ± SEM (*n* = 3 for each group). ns, no statistical significance, * *p* < 0.05, ** *p* < 0.01, LPS versus EcN + LPS.

It is evidenced in Figure 5A–F that there was a significant reduction in the concentrations of acetic, propionic, butyric, and isovaleric acids in mouse feces in the group subjected to LPS treatment. In contrast, pre-treatment with EcN was found to substantially restore the levels of acetic, butyric, and isovaleric acids.

We proceeded to investigate the expression of GPR41 and GPR43 in colonic tissues, which showed a pronounced reduction in the protein expression of both GPR41 and GPR43 in the LPS-exposed group when compared to the control group (*p* < 0.05). The introduction of EcN before LPS treatment led to a significant upregulation in the expression of these receptors, as depicted in Figure 5G,H, also with statistical significance (*p* < 0.05).

### 3.4. Acetate and Butyrate Increased the Expression of Expression of Tight Junction Proteins and GPRs in Caco-2 Monolayer Colonic Intestinal Epithelium Models

**Figure 6:**
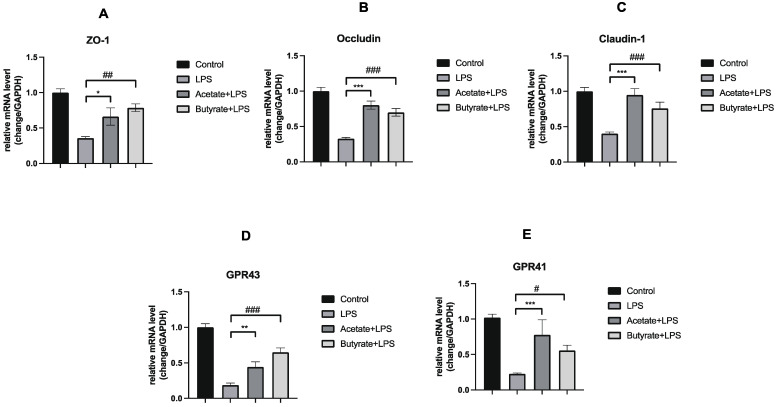
Supplement of acetate and butyrate on the expression of ZO-1 (**A**), Occludin (**B**), Claudin-1 (**C**), GPR43 (**D**), and GPR41 (**E**) in the Caco-2 monolayer. Control, control group; LPS, LPS group; Acetate + LPS, Acetate + LPS group Butyrate + LPS, Butyrate + LPS group. Data are expressed as the means ± SEM (*n* = 3 for each group). * *p* < 0.05, ** *p* < 0.01, *** *p* < 0.01, LPS versus Acetate + LPS. # *p* < 0.05, ## *p* < 0.01, ### *p* < 0.01, LPS versus Butyrate + LPS.

To elucidate the potential of EcN in augmenting the intestinal barrier primarily via the modulation of SCFAs, we conducted an experiment where Caco-2 monolayer cells were exposed to acetate and butyrate for 24 h, given our findings of EcN for the intestinal production of acetic acid and butyric acid increased. Subsequently, we assessed the influence of intestinal bacteria supernatant from different groups on the mRNA levels of tight junction proteins (ZO-1, Occludin, and Claudin-1) and GPRs (GPR41 and GPR43) within the Caco-2 cell monolayers. Our results indicated a marked reduction in the mRNA levels of these proteins after a 24 h exposure to LPS. Notably, the application of acetate and butyrate effectively counteracted the LPS-induced downregulation of ZO-1, Occludin, and Claudin-1 (Figure 6A–C). Acetate and butyrate also reversed the LPS-induced decline in GPR expression (Figure 6D,E).

This suggests that EcN may play a pivotal role in intestinal barrier function, potentially through the modulation of acetic acid and butyric acid and the activation of GPR41/43 receptors.

## 4. Discussion

The probiotic effects of EcN on enteritis have been well documented [5,22,23]. The purpose of this study was to focus on the effects of EcN on intestinal bacteria and intestinal SCFAs. Our research introduces a novel perspective by examining the modulatory role of EcN on the intestinal microbiome and its direct impact on SCFA levels, which are pivotal in maintaining intestinal homeostasis. By focusing on these aspects, our study contributes fresh insights into the mechanisms underlying EcN’s protective effects on the intestinal barrier.

The role of intestinal bacteria in intestinal inflammation is pivotal, as they engage closely with the host primarily through the exchange of small molecule metabolites to avert the onset and progression of disease [24,25]. Therefore, the effect of EcN on the intestinal biological barrier, i.e., the intestinal flora, is also of concern, in addition to the effect on the mechanical barrier. The results of 16S rRNA sequencing gave us the answer. *Bacteroidetes* and *Firmicutes*, the dominant bacteria that produce SCFAs in the gut, account for about 20% and 60% of the total intestinal flora, respectively. The content of SCFAs produced by *Proteobacteria* and *Actinobacteria* is relatively low, 5%~10% and 3%, respectively [26]. Our research has revealed that pre-treatment with EcN can significantly increase the abundance of Bacteroidetes and Firmicutes in septic shock mice; this intervention not only enhances the functionality of the intestinal barrier but also positively modulates the composition of the gut microbiota. It was reported that bacteria of the *Bacteroidetes* phylum produce high levels of acetate, whereas bacteria of the *Firmicutes* phylum produce high amounts of butyrate [27]. In addition, after the pre-treatment of EcN, the abundance of *f_Marinifilaceae*, *g_Muribaculaceae_g_norank*, *p_Bacteroidota*, and *o_Bacteroidales* had been raised. Among them, *f_Muribaculaceae* has been considered a protective factor against sepsis-induced liver injury [28]. *G_Muribaculaceae_g_norank* is famous for its function of improving the intestinal barrier [29]. *P_Bacteroidota* is associated with many health benefits, including downregulation of inflammation in the gut [30]. Furthermore, *f_Muribaculaceae* has been found to produce butyrate and *p_Bacteroidota* can be linked with acetic acid production [31]. Combined with the results of 16S rRNA, we can see that these data suggest that EcN acts as a key regulator to inhibit the colonization of harmful microorganisms, promote the colonization of probiotics, and beneficially promote two vital types of SCFA production.

SCFAs are found to be involved in systemic autoimmune responses and inflammatory processes and relieve intestinal inflammation by inhibiting the secretion of pro-inflammatory factors, reducing inflammatory responses and restoring intestinal barrier function [15,32,33]. Acetic acid, propionic acid, and butyric acid are the most common short-chain fatty acids in the gut, which has also been confirmed in our mouse experiments. In the following discussion, we will focus on the effects of these three short-chain fatty acids on the intestinal barrier. In our study, pre-treatment with EcN effectively increased the production of acetic acid and butyric acid, which suggests that EcN may protect the intestinal barrier by increasing the abundance of intestinal flora that produce acetic and butyric acid. Acetic acid was found to act as a protective endothelial barrier in LPS-induced animal models [34]. Butyric acid has long been known to have beneficial effects on the intestinal barrier [35]. It is particularly important to emphasize that the probiotic EcN 1917 has been designed to synthesize butyric acid, so that butyric acid is continuously produced in the intestines of mice with colitis to improve the intestinal microenvironment and reduce inflammation [36,37]. This suggests that EcN, in addition to promoting butyric acid production on its own, could serve as an excellent engineering site for in vivo therapy. The application of this engineered bacteria to sepsis patients may be a good research direction in the future. Propionic acid has also been observed to improve the intestinal barrier by enhancing the epithelial barrier and mucus barrier, but this finding was not seen in our study, possibly related to the smaller effect of EcN on propionic acid-producing gut microbes [38].

Previous research has demonstrated that the activation of GPRs can mitigate inflammation observed in the intestine and even in tumors, thereby promoting gut homeostasis induced by dietary fiber [39,40,41,42,43]. Mice lacking GPR43 exhibited exacerbated symptoms of both acute and chronic colitis [44]. The KEGG pathway with the most obvious enrichment of EcN treatment is membrane transport, which also indicates that EcN may be related to seven transmembrane GPRs. Our results showed that EcN treatment promoted the expansion of SCFA-producing bacteria, significantly restored the normal fecal levels of acetic acid and butyric acid, and upregulated the expression of GPR41/43, receptors of SCFAs, in colonic tissues of mice with colitis. At the same time, the supplementation experiment also found that acetate and butyrate could significantly increase the mRNA level of GPRs. These results suggested that EcN can prevent LPS-induced sepsis in mice by altering intestinal microbiota to increase the production of SCFAs, which may be related to the activation of GPR41/43 by SCFAs.

In conclusion, this study demonstrates the protective effect of EcN on intestinal barrier damage and uncovers the potential mechanism of EcN’s possible protective effect from the perspective of intestinal microecology and the SCFAs/GPRs pathway.

## Data Availability

The data presented in this study are available on request from the corresponding author due to privacy.
